# Blue Laser Irradiation Decreases the ATP Level in Mouse Skin and Increases the Production of Superoxide Anion and Hypochlorous Acid in Mouse Fibroblasts

**DOI:** 10.3390/biology11020301

**Published:** 2022-02-12

**Authors:** Eiko Nakayama, Toshihiro Kushibiki, Yoshine Mayumi, Ryuichi Azuma, Miya Ishihara, Tomoharu Kiyosawa

**Affiliations:** 1 Department of Plastic Surgery, National Defense Medical College, Saitama 3598513, Japan; azuma@ndmc.ac.jp (R.A.); xoo@ndmc.ac.jp (T.K.); 2 Department of Medical Engineering, National Defense Medical College, Saitama 3598513, Japan; toshi@ndmc.ac.jp (T.K.); yoshine@ndmc.ac.jp (Y.M.); kobako@ndmc.ac.jp (M.I.)

**Keywords:** photobiomodulation, laser, ATP, reactive oxygen species, reactive nitrogen species

## Abstract

**Simple Summary:**

Photobiomodulation studies have reported that blue light irradiation induces the production of reactive oxygen species. We examined the effect of blue laser (405 nm) irradiation on ATP level in the skin and measured the types of reactive oxygen species and reactive nitrogen species. The decrease in the skin ATP level due to blue light irradiation may be caused by oxidative stress due to the generation of reactive oxygen species. These findings highlight the need to consider the effects on the skin when performing photobiomodulation treatment using blue light.

**Abstract:**

Photobiomodulation studies have reported that blue light irradiation induces the production of reactive oxygen species. We investigated the effect of blue laser (405 nm) irradiation on the ATP levels in mouse skin and determined the types of reactive oxygen species and reactive nitrogen species using cultured mouse fibroblasts. Blue laser irradiation caused a decrease in the ATP level in the mouse skin and triggered the generation of superoxide anion and hypochlorous acid, whereas nitric oxide and peroxynitrite were not detected. Moreover, blue laser irradiation resulted in reduced cell viability. It is believed that the decrease in the skin ATP level due to blue light irradiation results from the increased levels of oxidative stress due to the generation of reactive oxygen species. This method of systematically measuring the levels of reactive oxygen species and reactive nitrogen species may be useful for understanding the effects of irradiation conditions.

## 1. Introduction

Under appropriate conditions, visible to near-infrared light irradiation exerts wound-healing, anti-inflammatory, anti-edema, and hair growth-promoting effects [1]. This phenomenon is called photobiomodulation (PBM). Light sources with wavelengths in the 400 nm range are used to treat psoriasis vulgaris, prurigo vulgaris, and atopy, as well as for tooth bleaching and restoration procedures involving composite resin [2,3,4]. In the treatment of acne vulgaris caused by *Cutibacterium acnes*, coproporphyrin III, a metabolite of *C. acnes*, acts as a photosensitizer. Reactive oxygen species (ROS) generated by irradiation with blue light can treat acne by sterilization [5]. The peak absorption of coproporphyrin III occurs at 415 nm and is effective in the presence of blue light [6]. Considering the therapeutic effects of blue light irradiation on acne, many practitioners perform irradiation for 10–20 min at an intensity of 30–90 mW/cm^2^ [5]. Therefore, a certain irradiation level is required to obtain effective therapeutic outcomes. However, compared to ultraviolet light, visible light may be harmless to living organisms, and its effect on healthy tissues has not been extensively examined [7].

Some studies have reported that ROS and reactive nitrogen species (RNS) are produced in response to PBM [8]. ROS are produced in response to large doses of light, particularly blue light [9,10]. Previous studies have attempted to identify the ROS and RNS types that are produced in response to blue light [11,12]. Indeed, high ROS and RNS levels may be harmful to living organisms. Excessive ROS and RNS levels can permanently damage the DNA, resulting in aging and cancer [13]. Although the underlying mechanisms remain unknown, both ROS and RNS appear to play important roles in PBM [14]. ROS is a generic term for various molecules and free radicals (chemical species with one unpaired electron) that are derived from molecular oxygen. ROS comprise oxygen free radicals, such as the superoxide anion (O_2_^−^•) and hydroxyl radical (•OH), and non-radical oxidants, such as hydrogen peroxide (H_2_O_2_), hypochlorous acid (HClO), and singlet oxygen (^1^O_2_) [15]. •OH is highly reactive and acts very close to the generation site, whereas O_2_^−^• and H_2_O_2_ are less reactive [15]. RNS includes the relatively unreactive nitric oxide (NO•); its derivative, peroxynitrite (ONOO^−^), is a strong oxidant that can damage several biomolecules [15]. ROS and RNS are by-products of PBM and important second messengers [16]. ROS cause an increase in the intracellular redox potential and activates several intracellular signal transduction pathways, such as nucleic acid synthesis, protein synthesis, enzyme activation, and cell-cycle progression [14,17]. These pathways involve transcription factors, such as nuclear factor-κB (NF-kB) and activator protein 1 (AP-1) [18]. NF-kB, a redox-regulated transcription factor, is a protein complex with multiple functions in the immune, inflammation, cell proliferation, and survival responses [17]. It induces the expression of inflammatory cytokines, such as interleukin (IL)-1, IL-2, IL-6, IL-8, IL-12, tumor necrosis factor-α, and interferon-γ, and can regulate the cellular immune, inflammatory, stress, proliferation, and apoptotic responses [19]. In our previous study, mouse skin irradiation using a 415 nm light-emitting diode (LED) caused dermal fibroblast apoptosis (Supplementary Figure S1). To better understand these changes caused by ROS, a detailed study of the types and amounts of ROS generated by PBM is required.

Numerous fluorescent probes have been developed recently and are reported to distinguish the various ROS and RNS types produced in the cells. With the advent of these fluorescent probes, the process of detection has become easier, more detailed, and more intuitive. Therefore, measuring the ROS and RNS generated during the process could enable the evaluation of the reaction pathway induced by these species, which could aid in elucidating the underlying mechanisms of PBM.

To our knowledge, this is the first study to investigate the effect of blue laser irradiation on the tissues by evaluating the changes in the ATP level in the skin and to perform a detailed investigation of the generated ROS. Changes in the skin ATP levels in mice irradiated with a 405 nm blue laser (precisely, blue-violet) were measured using a firefly luciferase luminescence assay. Furthermore, we aimed to identify the types of ROS and RNS produced in response to blue laser irradiation in fibroblasts using multiple fluorescent probes. We used mouse fibroblasts because irradiation with blue light can damage the deep layers of the skin. Several ROS and RNS types were systematically measured using the same cells under the same experimental conditions.

## 2. Materials and Methods

### 2.1. Measurement of the ATP Levels in Mouse Skin

All animal experiments were conducted in compliance with the ARRIVE guidelines and in accordance with the relevant guidelines and regulations of National Defense Medical College. The study protocol was approved by the Ethics Committee for Animal Experiments of National Defense Medical College (approval number: 19010). The abdomen of each C57BL/6 mouse (Japan SLC Inc., Hamamatsu, Japan) was shaved a day prior to the experiment. The mice were anesthetized using 2% isoflurane (MSD Animal Health, Kenilworth, NJ, USA); the mice were placed in the supine position, and the abdomen was irradiated using a blue laser (100 or 30 mW/cm^2^ for 20 min). The irradiation conditions were based on those used in clinical practice for PBM using blue light [20,21]. Skin tissues (5 × 5 mm) were collected immediately after irradiation. The skin tissues on the opposite side of the same mice were considered the non-irradiated samples (control group). Tissue ATP assay Kit (TOYO B-Net, Tokyo, Japan) was used to evaluate the ATP levels in the mouse skin. The mouse skin tissues were homogenized using PBS containing 0.1% Triton X-100 (MP Biomedicals, Santa Ana, CA, USA) as a homogenate buffer. Subsequently, supernatants were collected after centrifuging at 15,000 rpm (174 × 100 g) for 5 min at 4 °C. The samples were processed according to the manufacturer’s instructions, and luminescence levels were analyzed using a microplate reader (SpectraMax iD5; Molecular Devices, San Jose, CA, USA) to measure the ATP levels. To correct for variations in tissue volume, the protein content of each sample was measured using a TaKaRa BCA protein assay kit (item no. T9300A; TaKaRa Bio, Shiga, Japan), and the ATP levels were normalized to the protein content. The ATP levels are expressed as nmol/mg protein.

### 2.2. Cell Culture

L929 (American Type Culture Collection, Manassas, VA, USA) mouse fibroblasts of the subcutaneous connective tissue origin were used in this study. The cells were cultured in Dulbecco’s modified Eagle medium (DMEM; Thermo Fisher Scientific, Waltham, MA, USA) containing 10% fetal bovine serum (FBS; Thermo Fisher Scientific), 100 units/mL penicillin, 0.25 μg/mL amphotericin B, and 0.1 mg/mL streptomycin (Antibiotic-Antimycotic; Thermo Fisher Scientific) at 37 °C in a 5% CO_2_ atmosphere. L929 cells were seeded on a 96-well clear bottom culture plate (item no. 3860-096; AGC Techno Glass, Shizuoka, Japan) at a density of 4 × 10^4^ cells/well, 1 day before light irradiation. For the 3-(4,5-dimethylthiazol-2-yl)-2,5-diphenyltetrazolium bromide (MTT) assay, similar seeding was performed at a density of 5 × 10^3^ cells/well, 1 day preceding light irradiation. At the same time, the medium was changed to one lacking phenol red.

### 2.3. Fluorescent Probes for Detecting ROS and RNS

The intracellular ROS and RNS levels were measured by fluorescence analysis using five fluorescent probes. The details of these probes are presented in Table 1.

The cells were incubated in the dark at 37 °C with each of the fluorescent probes at the appropriate concentration and duration in DMEM-lacking FBS, antibiotics, and phenol red. After the incubation period, the cells were washed twice with Dulbecco’s phosphate-buffered saline without calcium and magnesium (14190144; Thermo Fisher Scientific). Subsequently, the medium was changed to DMEM supplemented with FBS but lacking phenol red before the laser irradiations were performed.

### 2.4. Laser Irradiation

A blue laser (RGBLase LLC, Fremont, CA, USA) with adjustable power (10–2000 mW) at a wavelength of 405 nm (continuous wave) was emitted directly from the fiber end at a distance of 5 cm from the surface of the mouse’s abdomen. The irradiated area of the mouse’s abdomen was a circle (diameter: 12 mm).

In the experiment using fibroblasts, the irradiated area in each well was 32.7 mm^2^. The cells were irradiated in the dark using a blue laser with a fiber attached to the bottom of the culture plate.

### 2.5. Fluorescence Analyses

In our previous studies, we demonstrated that irradiation with visible light at an intensity of 30 or 100 mW/cm^2^ significantly increased the ROS generation in different cell lines [22,23]. Moreover, a radiation intensity of 30–90 mW/cm^2^ was found to be effective for the treatment of acne vulgaris [4]. In this research, dose selection was based on our previous research [22]. The fluorescent probe incorporated L929 cells that were irradiated with a 405 nm laser, from the bottom of each well, at 100 or 30 mW/cm^2^ for 60 or 180 s. Fluorescent images were acquired using a fluorescence microscope (BZ-9000; Keyence, Osaka, Japan) pre- and post-laser irradiations. The cells were washed with PBS, treated with trypsin EDTA (0.25%) (Thermo Fisher Scientific) for dissociation, and subjected to flow cytometry (BD FACSCanto™ II; Becton, Dickinson and Company, Franklin Lakes, NJ, USA). The samples acquired for the fluorescence microscopy images were different from those that were used for flow cytometry. We used flow cytometry to measure the basal fluorescence of unstained cells and positive controls (Table 1). The mean fluorescence intensity (MFI) was determined for each cell group by analyzing >1 × 10^4^ cells in each group. The MFI ratio was calculated by dividing the MFI of irradiated cells (designated as irradiated MFI) by that of non-irradiated cells on the same day (designated as control MFI) as follows:

MFI ratio= (Irradiated MFI)/(Control MFI)

The measurements were performed on the same day due to daily MFI variation based on the cell state, including the degree of confluence and fluorescent probe uptake.

### 2.6. MTT Assay

An MTT cell count kit (item no. 23506-80; Nacalai Tesque, Inc., Kyoto, Japan) was used to assess cell viability following blue laser irradiation. Cells were seeded at 5 × 10^3^/well in 96-well plates and irradiated using a 405 nm laser from the bottom of each well at 100 or 30 mW/cm^2^ for 60 or 180 s. The cells were subsequently left in the dark at 37 °C for 24 h. After incubation, 10 μL of MTT solution was added to each well, and the absorbances after 4 h were measured using the 450 nm filter of a microplate reader (SpectraMax iD5; Molecular Devices Corp., San Jose, CA, USA).

### 2.7. Statistical Analysis

Statistical analyses were performed using JMP Pro 15 software (SAS Institute Japan; Tokyo, Japan). To measure the ATP levels of the mouse skin, the data were analyzed using the unpaired two-tailed t-test. The MFI ratios to detect ROS and RNS were determined using seven independent experiments; the results were analyzed using a sign test. The MTT assay was performed in five independent experiments. A *p*-value < 0.05 was considered statistically significant.

## 3. Results

### 3.1. Blue Laser Irradiation Reduced the ATP Levels in the Mouse Skin

**Figure 1:**
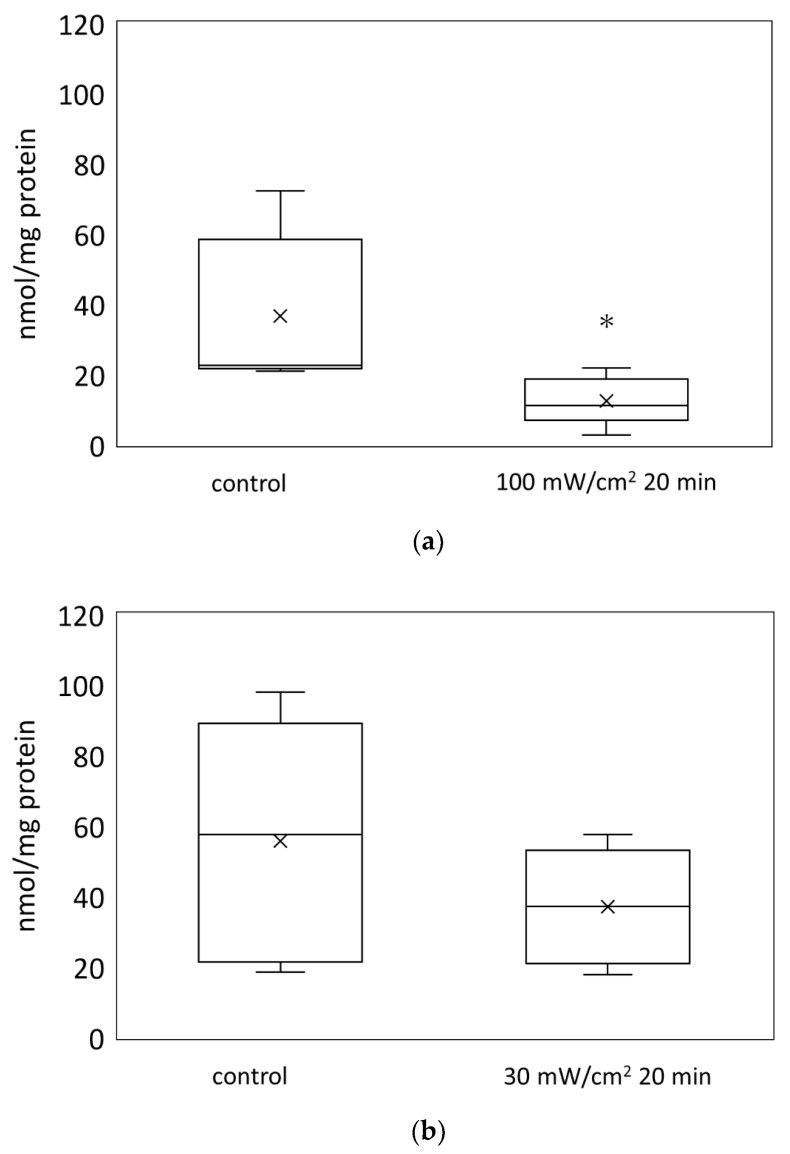
The ATP levels in the mouse skin after blue laser irradiation. The ATP levels in the mouse skin were measured using firefly luciferase luminescence assay. The irradiation conditions were (**a**) 100 mW/cm^2^, 20 min, 120 J and (**b**) 30 mW/cm^2^, 20 min, 36 J. * *p* < 0.05 against the control group.

The ATP levels in the mouse skin were measured using firefly luciferase luminescence assay. The skin ATP levels were significantly decreased after 20 min of irradiation at 100 mW/cm^2^ (Figure 1).

### 3.2. Blue Laser Irradiation Induces the Generation of O_2_^−^• and HClO, but Not the Generation of NO• and ONOO^−^

**Figure 2:**
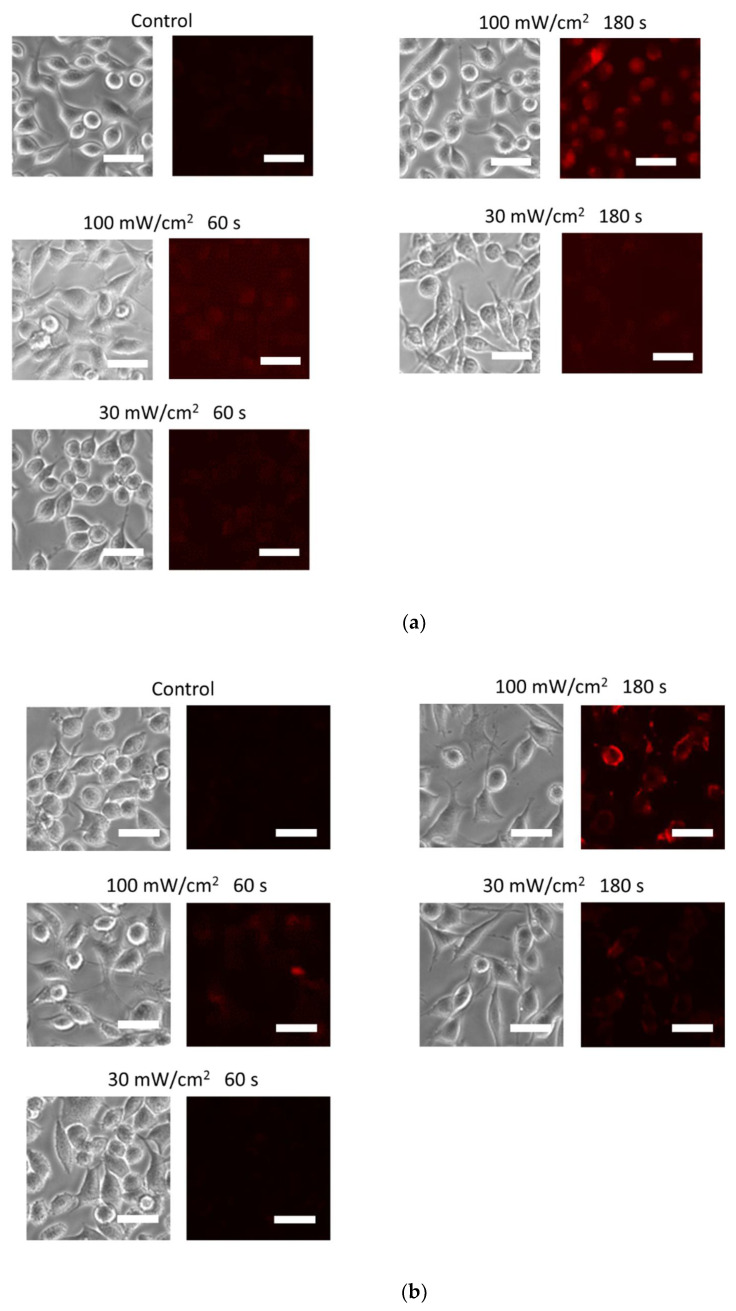

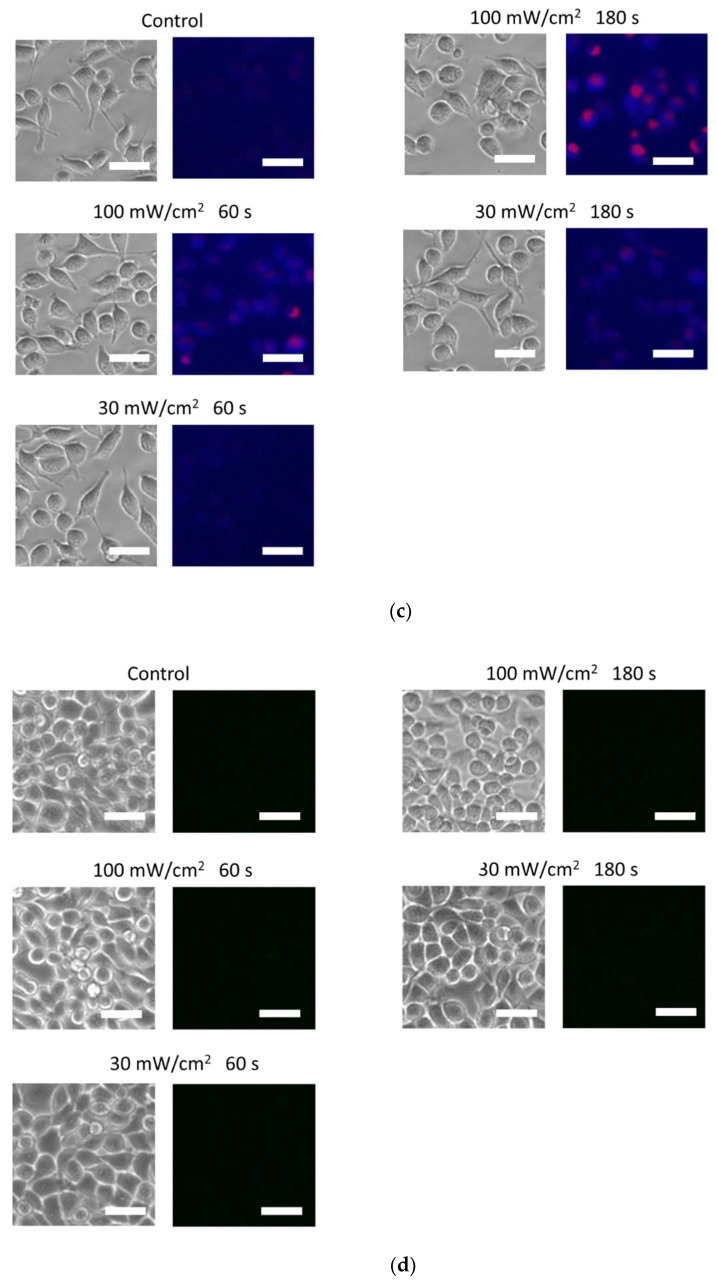

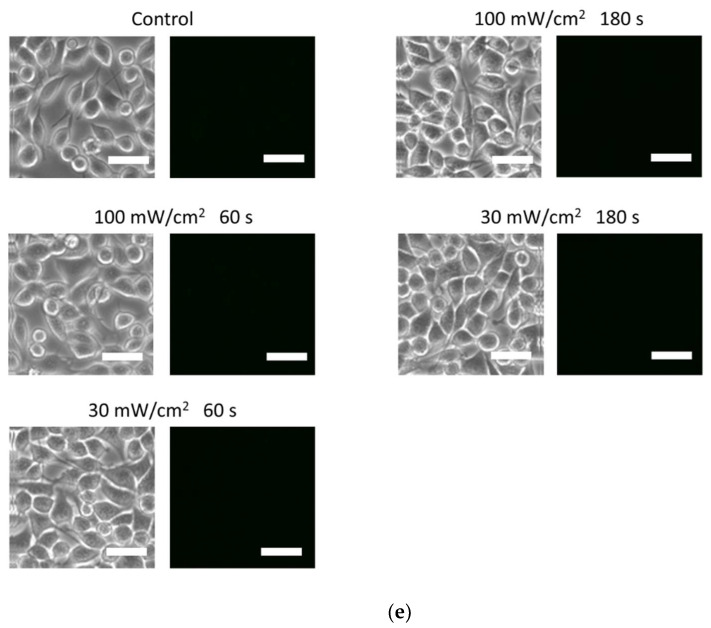
ROS and RNS generation after blue laser irradiation. Five fluorescent probes were incorporated into the L929 mouse fibroblasts. The cells were irradiated with a blue laser (405 nm), and the generation of ROS and RNS was evaluated using fluorescence microscopy. Phase-contrast microscopic images of the same sites are presented beside the fluorescent microscopic images. The fluorescent probes used were (**a**) OxiORANGE (detects •OH and HClO), (**b**) HySOx (detects HClO), (**c**) dihydroethidium (detects O_2_^−^•), (**d**) Nitrixyte Red (detects NO•), and (**e**) NiSPY-3 (detects ONOO^−^). The irradiation conditions were as follows: (1) control, no laser irradiation; (2) 100 mW/cm^2^, 180 s, 18 J; (3) 100 mW/cm^2^, 60 s, 6 J; (4) 30 mW/cm^2^, 180 s, 5.4 J; (5) 30 mW/cm^2^, 60 s, 1.8 J. Scale bar = 30 μm. •OH, hydroxyl radical; HClO, hypochlorous acid; O_2_^−^•, superoxide anion; NO•, nitric oxide; ONOO−, peroxynitrite.

First, we assessed the ROS and RNS generation in L929 cells with incorporated fluorescent ROS and RNS detection probes after irradiation with a 405 nm blue laser, using fluorescence microscopy. Fluorescence intensities of OxiORANGE, which detects •OH and HClO; HySOx, which detects HClO; and dihydroethidium, which relatively specifically detects O_2_^−^•, significantly increased after comparison with those before irradiation (Figure 2). The highest fluorescence intensity was observed for dihydroethidium with 100 mW/cm^2^ irradiation for 180 s. The fluorescence intensities of Nitrixyte Red, which detects NO•, and NiSPY-3, which detects ONOO^−^, remained unchanged following irradiation under the same conditions.

**Figure 3:**
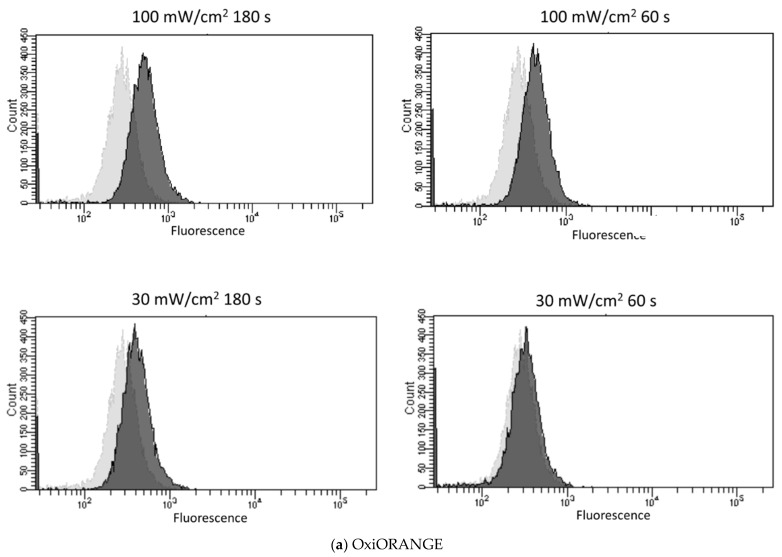

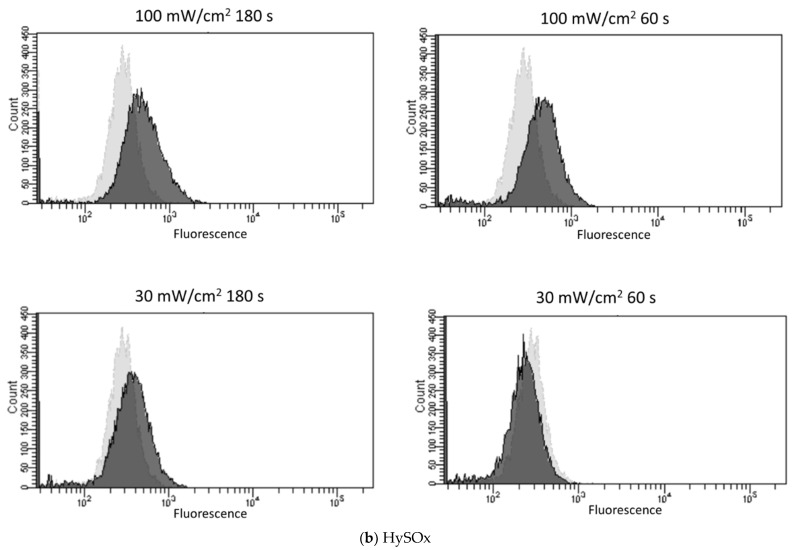

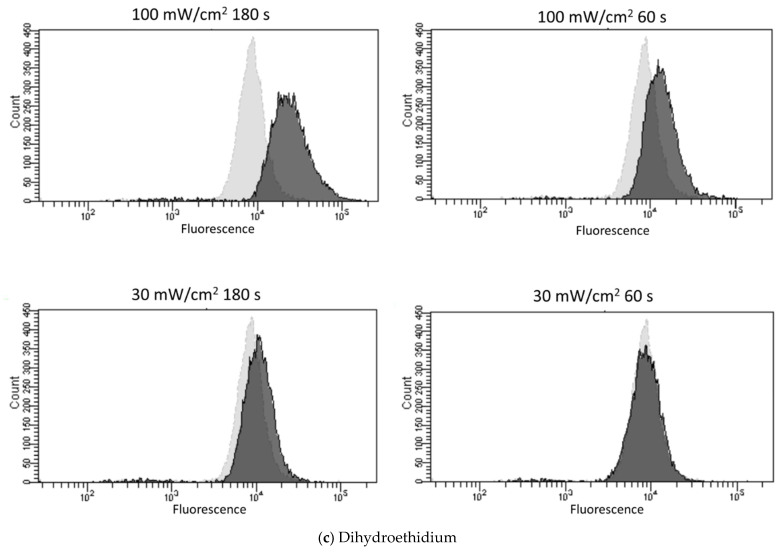

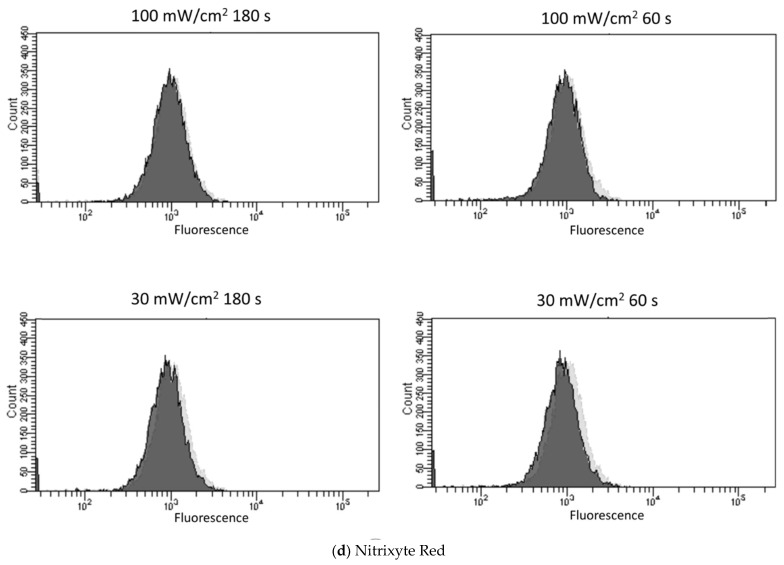

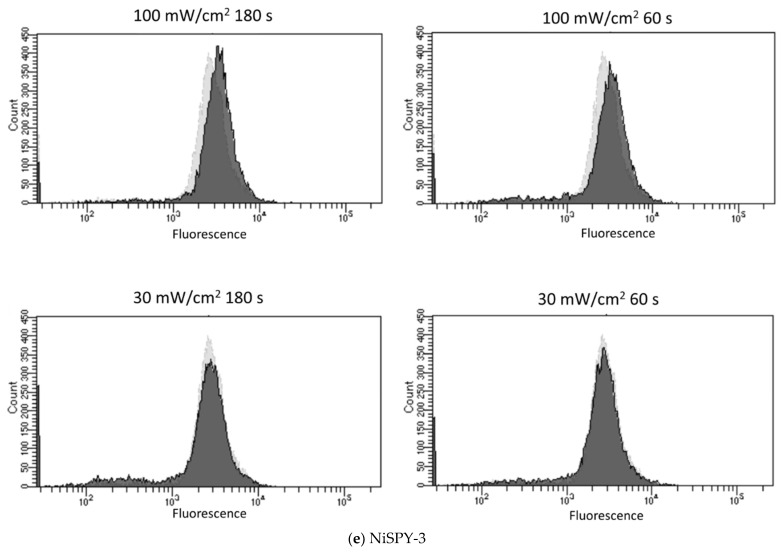
Representative histogram following blue laser irradiation obtained by flow cytometry. Five fluorescent probes were incorporated into the L929 mouse fibroblasts, and the cells were irradiated with a blue laser (405 nm). Each graph separately indicates the histogram for each fluorescent probe: (**a**) OxiORANGE (detects •OH and HClO), (**b**) HySOx (detects HClO), (**c**) dihydroethidium (detects O_2_^−^•), (**d**) Nitrixyte Red (detects NO•), and (**e**) NiSPY-3 (detects ONOO^−^). The irradiation conditions were as follows: (1) control, 100 mW/cm^2^, 180 s, 18 J; (2) 100 mW/cm^2^, 60 s, 6 J; (3) 30 mW/cm^2^, 180 s, 5.4 J; (4) 30 mW/cm^2^, 60 s, 1.8 J. Light and dark gray histograms correspond to the control and irradiation groups, respectively. •OH, hydroxyl radical; HClO, hypochlorous acid; O_2_^−^•, superoxide anion; NO•, nitric oxide; ONOO−, peroxynitrite.

Figure 3 shows a representative histogram of the change in the fluorescence intensity obtained by flow cytometry when the cells incorporating each fluorescent probe were irradiated with a blue laser under various conditions. The light and dark gray histograms correspond to the non-irradiated and irradiated groups, respectively. In OxiORANGE (detects •OH and HClO), HySOX (detects HClO), and dihydroethidium (detects O_2_^−^•), the peak of fluorescence intensity shifted mainly in the 100 mW/cm^2^ irradiated group. When using Nitrixyte Red (detects NO•) and NiSPY-3 (detects ONOO^−^), no obvious shifts in the fluorescence brightness were observed.

**Figure 4:**
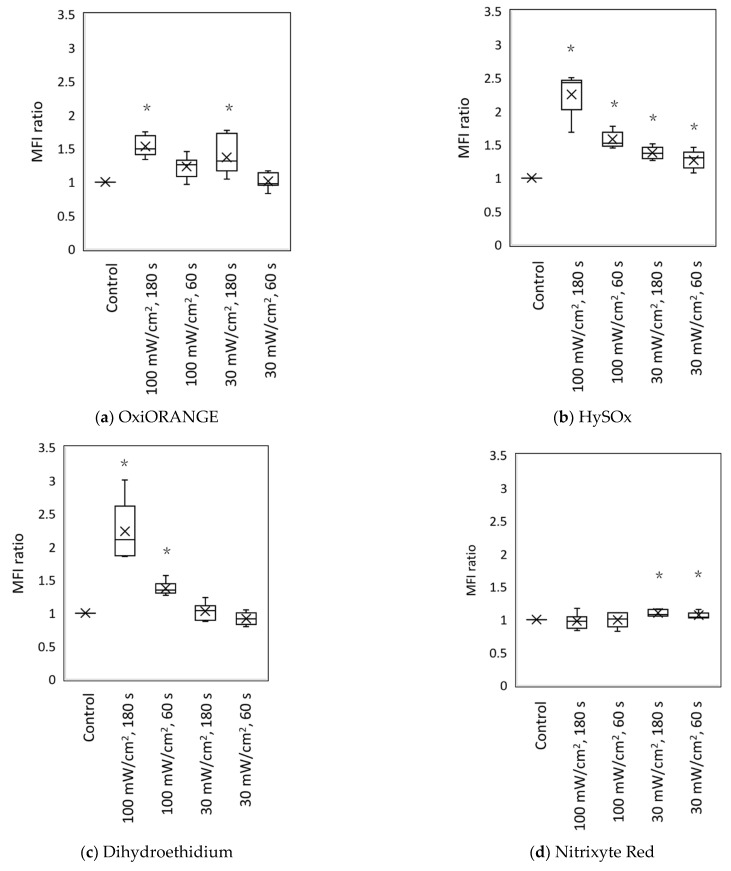

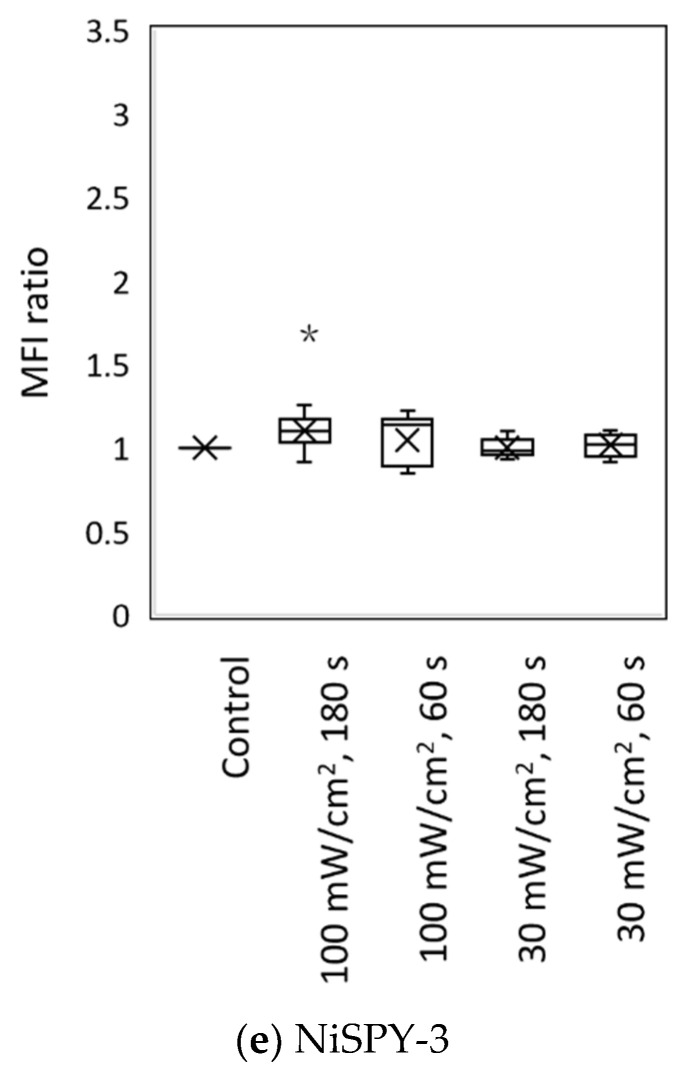
The MFI following blue laser irradiation. Five fluorescent probes were incorporated into the L929 mouse fibroblasts, and the cells were irradiated with a blue laser (405 nm). Each graph separately indicates the MFI ratio for each fluorescent probe: (**a**) OxiORANGE (detects •OH and HClO), (**b**) HySOx (detects HClO), (**c**) dihydroethidium (detects O_2_^−^•), (**d**) Nitrixyte Red (detects NO•), and (**e**) NiSPY-3 (detects ONOO^−^). The irradiation conditions were as follows: (1) control, 100 mW/cm^2^, 180 s, 18 J; (2) 100 mW/cm^2^, 60 s, 6 J; 30 mW/cm^2^, 180 s, (3) 5.4 J; 30 mW/cm^2^, 60 s, 1.8 J. The MFIs of the positive control for each fluorescent probe (Table 1; n ≥ 3) were as follows: (**a**) OxiORANGE, 2.73; (**b**) HySOx, 44.46; (**c**) dihydroethidium, 3.21; (**d**) Nitrixyte Red, 2.95; (**e**) NiSPY-3, 1.99. The MFIs of the negative control (unstained cells) (Table 1; n ≥ 3) were as follows: (**a**) OxiORANGE, 0.66; (**b**) HySOx, 0.99; (**c**) dihydroethidium, 0.09; (**d**) Nitrixyte Red, 0.90; (**e**) NiSPY-3, 1.02. The MFI was measured using flow cytometry; the MFI ratio was calculated by dividing the MFI of irradiated cells with that of non-irradiated cells. * *p* < 0.05. •OH, hydroxyl radical; HClO, hypochlorous acid; O_2_^−^•, superoxide anion; NO•, nitric oxide; ONOO−, peroxynitrite; MFI, mean fluorescence intensity.

Figure 4 presents the MFI ratios for each fluorescent probe, which was calculated from the MFI obtained using flow cytometry. For OxiORANGE (detects •OH and HClO), the MFI ratio significantly increased under the following irradiation conditions: (1) 100 mW/cm^2^, 180 s, and (2) 30 mW/cm^2^, 180 s (Figure 4a). For HySOx (detects HClO), the MFI ratio significantly increased under all irradiation conditions (Figure 4b), whereas for dihydroethidium (detects O_2_^−^•), the MFI ratio significantly increased under irradiation conditions of 100 mW/cm^2^ for 180 s and 100 mW/cm^2^ for 60 s (Figure 4c). For the Nitrixyte Red (detects of NO•), the MFI ratio slightly increased under irradiation conditions of 30 mW/cm^2^ for 180 s and 30 mW/cm^2^ for 60 s (Figure 4d). Finally, for the NiSPY-3 (detects of ONOO^−^), the MFI ratio slightly increased when irradiated at 100 mW/cm^2^ for 180 s (Figure 4e).

### 3.3. Irradiation of a Blue Laser Reduces Cell Viability of L929 Cells

**Figure 5:**
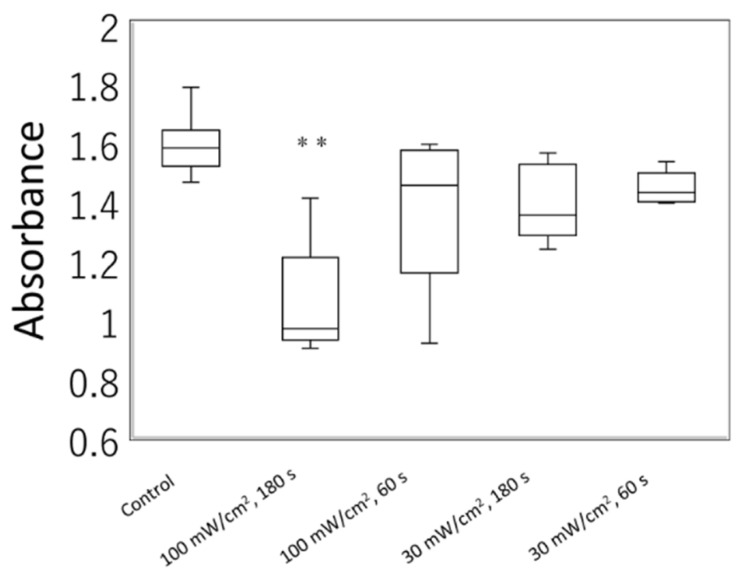
The cell viability assessment of irradiated cells. The 3-(4,5-dimethylthiazol-2-yl)-2,5-diphenyltetrazolium bromide (MTT) assay reagent was added at 24 h after the irradiation of L929 mouse fibroblasts with a blue laser of 405 nm, under various conditions (100 mW/cm^2^, 180 s, 18 J; 100 mW/cm^2^, 60 s, 6 J; 30 mW/cm^2^, 180 s, 5.4 J; 30 mW/cm^2^, 60 s, 1.8 J). Absorbance was measured at 4 h following MTT treatment. At least five independent experiments are presented. The results of the Kruskal–Wallis test followed by Dunnett’s multiple comparison tests showed a significant difference (*p* < 0.01) among the five groups. ** *p* < 0.01 against the control group.

Cell viability assessment of L929 cells irradiated with a blue laser under various conditions was performed as follows: 100 mW/cm^2^ for 180 s, 100 mW/cm^2^ for 60 s, 30 mW/cm^2^ for 180 s, and 30 mW/cm^2^ for 60 s. At 4 h following MTT treatment, we observed significantly reduced cell viability under 100 mW/cm^2^ for 180 s compared to that of the controls (Dunnett’s multiple comparison tests; Figure 5).

## 4. Discussion

As an indicator of oxidative stress, intracellular ATP concentration is associated with homeostasis of mitochondrial metabolism [24,25]. A decrease in the mitochondrial membrane potential may lead to a decrease in ATP production [26]. Thus, the decrease in the ATP level of the skin caused by blue laser irradiation may be attributed to mitochondrial damage caused by the ROS generation. In previous works, increases in ATP levels and other positive changes have been reported with low doses of blue light irradiation [27,28]. However, the irradiation conditions we employed in this experiment seemed to be more oxidatively stressful than those that could exert such a positive effect.

The mitochondrial respiratory chain constitutes the major intracellular ROS source in most tissues. The major ROS type that is generated in the mitochondria by univalent autoxidation of electron carriers is O_2_^−^•. Superoxide dismutase (SOD) catalyzes the dismutation of O_2_^−^•, thereby generating H_2_O_2_. Furthermore, H_2_O_2_ reacts with redox-active metals, such as iron, to produce •OH in a process called the Fenton/Haber–Weiss reaction [15,29]. •OH is a major cause of oxidative damage to DNA bases and is generated by various mechanisms. H_2_O_2_ can also react with a chloride anion to form HClO, a reaction that requires myeloperoxidase. The latter is abundantly present in neutrophils, but it is also found in neuronal cells under certain conditions. HClO is a major end product of neutrophil respiratory burst. Endogenous HClO is a powerful natural oxidant that can react with proteins, DNA, RNA, fatty acids, and cholesterol, and plays vital physiological roles in living organisms [30]. To our knowledge, this is the first study to report blue light irradiation-induced HClO production in fibroblasts.

Several studies have reported NO• generation by blue laser or red to near-infrared laser irradiation [31,32,33]. Irradiation of human skin with blue light (420–453 nm) at 58 mW/cm^2^ for ≥2 min was shown to induce non-enzymatic nitric oxide generation from photo-labile nitric oxide derivates in vitro and in vivo [34]. Lindgard et al. reported that the irradiation of human monocytes with a 634 nm LED at 35.7 W/cm^2^ for 5 min generated NO• [31]. Moreover, near-infrared radiation has been shown to dissociate NO• from cytochrome c oxidase, a key chromophore in PBM, and increase the intracellular NO• levels in cardiomyocytes and HeLa cells [35]. Cytochrome c oxidase or Complex IV is a respiratory electron transport chain component. The application of light, particularly PBM, increases ATP production and electron transport [36]. Another RNS, ONOO^−^, is produced by the diffusion-controlled reaction of NO•and O_2_^−^• [37]. ONOO^−^ provides several beneficial effects and plays an auxiliary regulatory role in the immune system; however, its overproduction exerts harmful effects on the cell metabolism by inducing lipid peroxidation, DNA damage, and mitochondrial damage [16]. However, in our study, under irradiation conditions, we could not detect any RNS type, which could be attributed to the lower wavelength (405 nm) used compared with those of previous studies (>420 nm).

Blue light intracellular photoreceptors, including opsins, flavoproteins, and porphyrins, play important roles in the ROS and RNS generation. Opsin receptors (OPNs) are G protein-coupled receptors excited by blue or green light [28]. Human melanocytes and keratinocytes express four other opsins (i.e., OPN1-SW, OPN2, OPN3, and OPN5) [38]. OPN3 has also been detected in human fibroblasts [39]. Opening of light-gated ion channels, such as the transient receptor potential (TRP) family of calcium channels, is one of the best-defined signaling events that occurs after light-activation of opsins [40]. TRP activation results in nonselective permeabilization of the plasma membrane to calcium, sodium, and magnesium, possibly causing an increase in the intracellular calcium and consequently increasing the ROS and RNS, particularly NO• [41]. Flavoproteins contain a riboflavin derivative (flavin adenine dinucleotide or flavin mononucleotide) as a prosthetic group and can be excited by blue light (400–500 nm) [42]. O_2_^−^• is generated by the photolytic intermediate of riboflavin, flavin mononucleotide, and flavin adenine dinucleotide [43]. Moreover, porphyrins have been observed in the human body [44]. Porphyrins have an absorption peak of approximately 418 nm, can absorb blue light, and generate ^1^O_2_ when excited to the triplet state [45]. Porphyrin precursors, such as 5-aminolevulinic acid, have been used in photodynamic therapy to kill cancer cells and vascular endothelial cells [46]. These reactions of porphyrins to blue light have been shown to account for the antibacterial properties of PBM [47]. Moreover, a wavelength of approximately 418 nm is commonly used for acne treatment. Despite the important role of these photoreceptors, we could not identify the specific photoreceptors involved in ROS generation under the experimental conditions. Therefore, further investigation is required.

Cells possess various enzymatic systems for detoxifying ROS. SOD catalyzes the conversion of O_2_^−^• to O_2_ and H_2_O_2_. Although O_2_^−^• is not very reactive, it reacts with the surrounding components to generate more reactive ROS, such as ONOO^−^ and H_2_O_2_. O_2_^−^• oxidizes iron–sulfur clusters of proteins (such as the cytoplasmic enzymes, aconitase, and fumarase in the citric acid cycle), thereby degrading cluster iron and losing catalytic iron, resulting in enzyme inactivation [48]. Iron atoms released in this process directly reduce H_2_O_2_ to produce more toxic •OH. The majority of mitochondrial H_2_O_2_ is generated by the disproportionation of O_2_^−^• via MnSOD, which regulates the O_2_^−^• levels and establishes the intracellular H_2_O_2_ flux [49]. Therefore, SOD plays an integral role in controlling ROS and RNS concentrations in vivo [29]. We measured the total SOD activity after providing blue laser irradiation (Supplementary Figure S2). SOD activity is altered in response to various stimuli, but the time required for SOD activation varies [50,51].

In our study, the cells that received dihydroethidium presented significantly increased fluorescence with 405 nm laser irradiation at 100 mW/cm^2^. Dihydroethidium reacts with O_2_^−^• to produce 2-hydroxyethidium, a highly specific red fluorescence product. However, biological systems can react with other substances to form another red fluorescence product, ethidium. Moreover, dihydroethidium alone cannot completely identify superoxide [52]. Therefore, we should consider the involvement of various other ROS. Dihydroethidium does not react with singlet oxygen or H_2_O_2_; however, this issue requires further investigation. Furthermore, this probe may underestimate O_2_^−^• formation due to its short half-life and high rate of occurrence of the disproportionation reaction [53]. Therefore, we cannot refute possible O_2_^−^• generation below the detection limit under 30 mW/cm^2^ irradiation. ROS may act as signaling molecules at low concentrations and exhibit cytotoxic properties at high concentrations. A previous study involving irradiation of human fibroblasts using 410–480 nm blue light revealed that higher irradiation doses induced higher cytotoxicity, as evidenced by cell growth suppression [34]. Similarly, we observed a significant decrease in the cell viability assessment under 100 mW/cm^2^ irradiation for 180 s. Therefore, the 405 nm, 100 mW/cm^2^ laser irradiation for 180 s, which was provided in our experiment, may have caused certain damage.

Several studies have described blue light irradiation as a relatively reliable treatment method for acne [2]. However, no consensus exists on the light source wavelength, duration, and intensity of irradiation. The experimental conditions in our study were consistent with those of our previous study that detected ROS using blue light [22]. Additionally, the irradiation intensity was inferred from clinical trials that attempted to treat acne and psoriasis (approximately 30–100 mW/cm^2^). Furthermore, although treatments for acne vulgaris and psoriasis require longer irradiation periods, the effects of longer irradiation in in vitro studies could be drastic due to system sensitivity; therefore, we applied a shorter treatment duration. Moreover, considering that the skin has multiple cells and components that interact with each other, this finding cannot simply be applied in vivo, as the study was conducted only on fibroblasts. We inferred that excessively intense blue light irradiation could cause oxidative skin damage. However, detailed studies involving animal and human disease models are required to investigate the irradiation conditions that could exert maximal therapeutic effects with minimal oxidative stress.

We demonstrated that prolonged exposure to 405 nm laser irradiation significantly decreased the ATP levels in mouse skin and stimulated the production of HClO and O_2_^−^•, whereas the generation of NO• and ONOO^−^ was limited. Previous PBM studies have demonstrated the types of ROS and RNS that occur in various cells and tissues [9,11,12,54]. However, unlike our experiment, no attempts were made to detect multiple ROS and RNS simultaneously and systematically. Nevertheless, it is difficult to directly compare research results with those of other studies because there are various parameters, including the provided wavelength, irradiation density, irradiation time, and pulse width. Therefore, as noted in our research, investigating multiple ROS and RNS concurrently under certain conditions elucidates the individuality of irradiation conditions.

This study may serve as a basis for future research aimed at elucidating the formation mechanisms and subsequent reaction pathways of ROS and RNS. Our findings also highlight that oxidative stress from over-irradiation with blue light can cause adverse effects, such as aging. Furthermore, they may aid in the development of PBM-based therapeutic strategies. Further studies to investigate the most effective and harmless blue light irradiation conditions (wavelength, intensity, and time) using animal and human disease models are currently being performed in our laboratory.

## 5. Conclusions

Blue laser irradiation reduced the ATP levels in mouse skin and triggered the generation of O_2_—and HClO. In addition, 405 nm blue laser irradiation at 100 mW/cm^2^ for 180 s decreased cell viability. These results suggest that caution is needed while using blue laser irradiation, as it could induce ROS production. Furthermore, our method of simultaneous measurement of ROS and RNS can be an appropriate experimental method to understand the characteristics of the complex irradiation conditions of PBM.

## Figures and Tables

**Table 1:** Fluorescent probes used in the study and the ROS and RNS detected by each fluorescent probe.

Fluorescent Probe	Excitation (nm)/Emission (nm)	Detectable ROS and RNS ^a^	Final Concentration	Incubation Time (min)	Positive Control Concentration	Supplier
OxiORANGE	543/577	•OH, HClO	1 μM	20	H_2_O_2_ 500 μM	Goryo Chemical, Hokkaido, Japan
HySOx	555/575	HClO	5 μM	30	NaOCl 5 μM	Goryo Chemical
Dihydroethidium	518/606	O_2_^−^•	10 μM	30	Antimycin A 10 mM	Thermo Fisher Scientific
Nitrixyte Red	630/660	NO•	1× ^b^	30	NONOate 50 mM	AAT Bioquest, Sunnyvale, CA, USA
NiSPY-3	490/515	ONOO^−^	10 μM	30	Peroxynitrite 20 µM	Goryo Chemical

^a^ •OH, hydroxyl radical; HClO, hypochlorous acid; O_2_^−^•, superoxide anion; NO•, nitric oxide; ONOO^−^, peroxynitrite. ^b^ The purchased stock solution was diluted 500 times.

## Data Availability

Data is contained within the article or Supplementary Material.

## Supplementary Materials

The following is available online at https://www.mdpi.com/article/10.3390/biology11020301/s1, Supplementary Figure S1: Apoptosis in mouse fibroblasts (a) Irradiated tissue and (b) non-irradiated tissue, Supplementary Figure S2: Superoxide dismutase (SOD) activity in irradiated cells.
