# Supplementary material for: Blue Laser Irradiation Decreases the ATP Level in Mouse Skin and Increases the Production of Superoxide Anion and Hypochlorous Acid in Mouse Fibroblasts
Source: Biology (Basel). 2022 Feb 12;11(2):301. doi: 10.3390/biology11020301 (PMC8869339; doi:10.3390/biology11020301)
Supplement: Supplementary file 1 [file biology-11-00301-s001.zip › biology-1567797-supplementary.pdf]

Supplementary Figure S1

1. Apoptosis in mouse fibroblasts

(a) Irradiated tissue

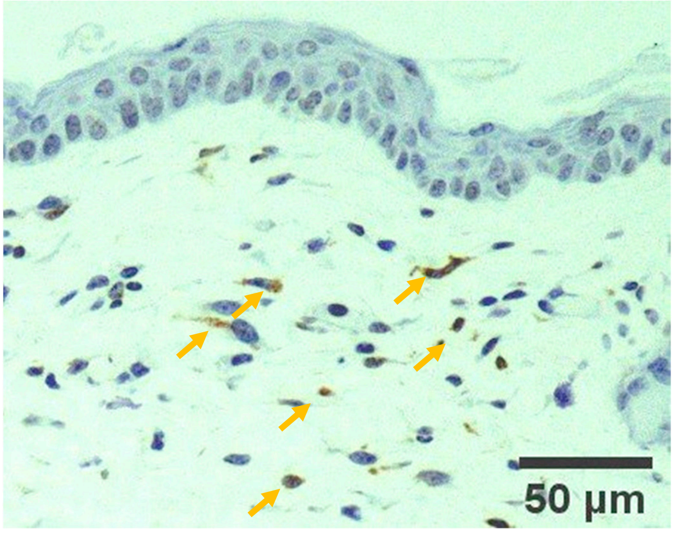

(b) Non-irradiated tissue

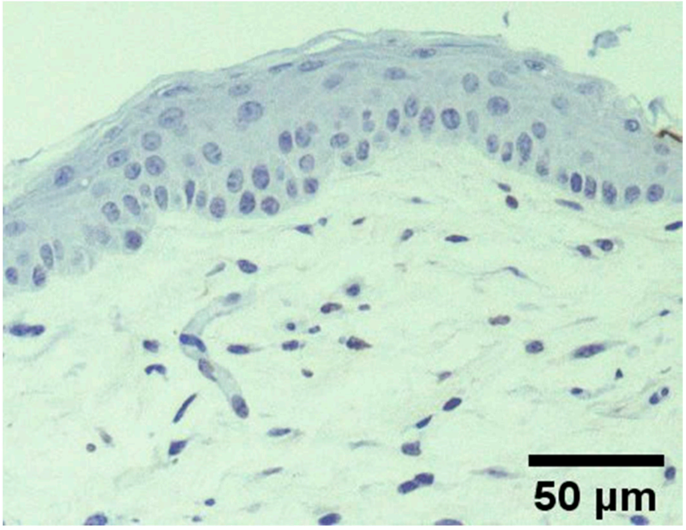

Figure S1. The abdomen of C57BL/6 mice was irradiated with a 415-nm LED at 100 mW/cm^2 for 20 min, and the pathological tissue was collected after 7 days

When a TUNEL staining was performed, TUNEL-positive cells (yellow arrows) were found in the epidermal cells and dermal fibroblasts of the LED-irradiated tissue (a), but not in the non-irradiated tissue (b).

Supplementary Figure 2

2. Superoxide dismutase assay

2.1 Material & Method

A superoxide dismutase (SOD) assay kit (Item No. 706002; Cayman Chemicals Inc., Ann Arbor, MI, USA) was used to evaluate intracellular SOD activity (U/mL). This assay utilizes tetrazolium salt to detect O_2^{-} produced by xanthine oxidase and hypoxanthine. A standard curve was generated with the known exogenous SOD included in the kit. Confluent cells were irradiated using a 405-nm laser from the bottom of each well, at either 100 or 30 mW/cm^2 for 60 or 180 s. Subsequently, the cells were incubated in the dark at 37°C for 30, 60, and 120 min, and were washed once with PBS and lysed with PBS-containing 0.1% Triton X-100 (MP Biomedicals, Santa Ana, CA, USA). Subsequently, supernatants were collected after centrifuging at 15 000 rpm (174 × 100 g) for 5 min at 4°C. Samples were processed according to the manufacturer's instructions and analyzed using a microplate reader (SpectraMax iD5; Molecular Devices, San Jose, CA, USA) equipped with a 450-nm filter to determine the SOD activity. To correct for variations in the number of lysed cells, the protein content in each sample was measured using a TaKaRa BCA protein assay kit (Item No, T9300A; TaKaRa Bio, Shiga, Japan), and the SOD activity was normalized to the protein content. Data were analyzed using the Kruskal-Wallis test, followed by the Dunnett's multiple comparison tests. A *P*-value of <0.05 was considered statistically significant.

2.2 Result

Irradiation with blue laser increases SOD activity in L929 cells

The L929 cells were irradiated with a blue laser under various conditions: 100 mW/cm^2 for 180 s, 100 mW/cm^2 for 60 s, 30 mW/cm^2 for 180 s, and 30 mW/cm^2 for 60 s. The SOD activity was measured before and after 30, 60, and 120 min of irradiation with no change observed under the irradiation conditions of 100 mW/cm^2 for 180 s, whereas under 100 mW/cm^2 for 60 s and 30 mW/cm^2 for 60 s conditions, a significant increase in the SOD activity was observed at 120 min following the irradiation (Figure 2).

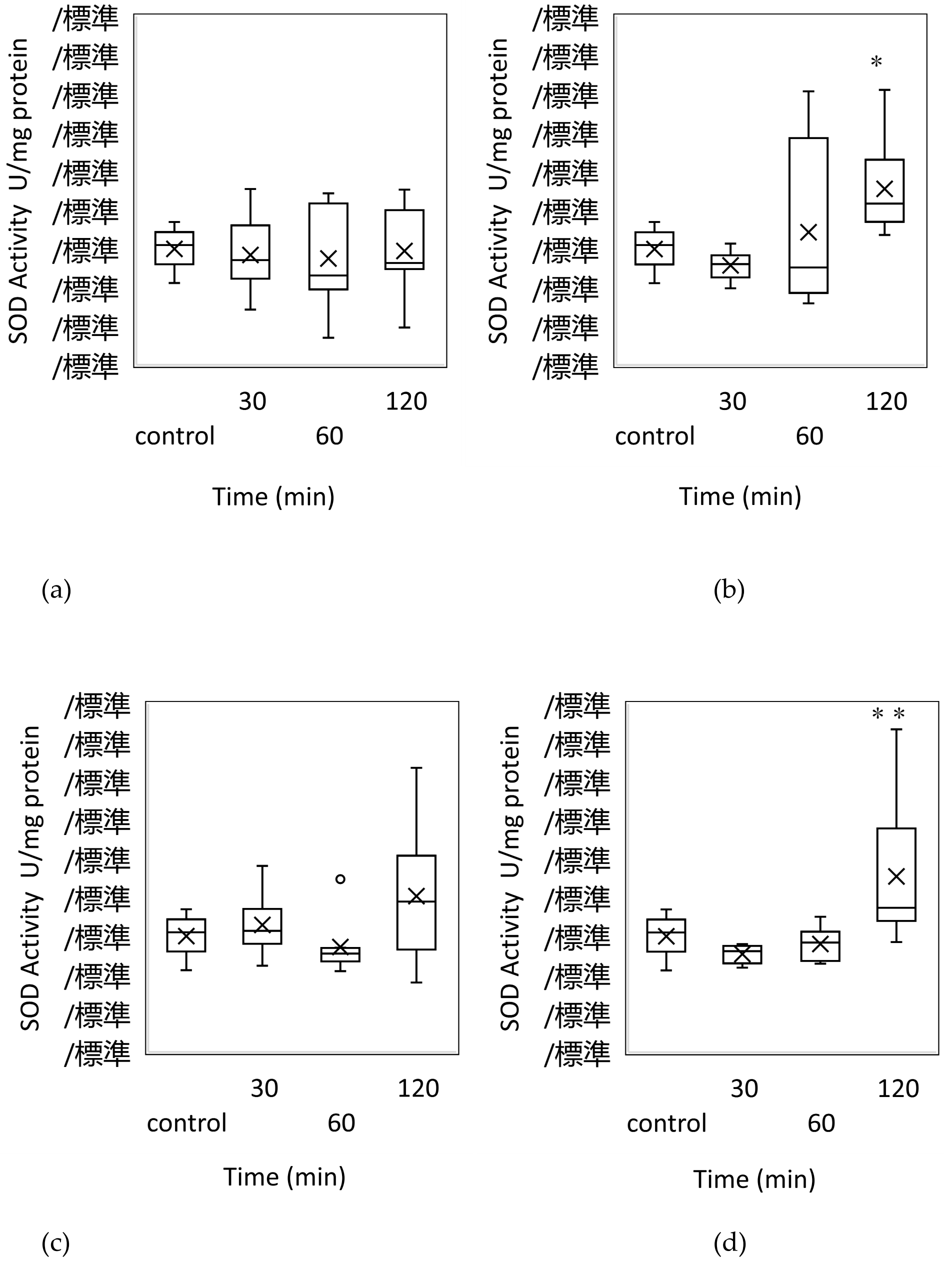

Figure S2. Superoxide dismutase (SOD) activity in irradiated cells

The SOD activity (U/mg protein) was measured before (control) and 30 min, 60 min, and 120 min after irradiating the L929 mouse fibroblasts with a 405-nm laser under the following conditions: (a) 100 mW/cm^2, 180 s, and 18 J; (b) 100 mW/cm^2, 60 s, and 6 J; (c) 30 mW/cm^2, 180 s, and 5.4 J; and (d) 30 mW/cm^2, 60 s, and 1.8 J. At least eight independent experiments are shown. *P<0.05, **P<0.01 against the control group.
